# Interleukin-17A Gene Haplotypes Are Associated with Risk of Premature Coronary Artery Disease in Mexican Patients from the Genetics of Atherosclerotic Disease (GEA) Study

**DOI:** 10.1371/journal.pone.0114943

**Published:** 2015-01-23

**Authors:** Gilberto Vargas-Alarcón, Javier Angeles-Martínez, Teresa Villarreal-Molina, Edith Alvarez-León, Rosalinda Posadas-Sánchez, Guillermo Cardoso-Saldaña, Julian Ramírez-Bello, Nonanzit Pérez-Hernández, Juan Gabriel Juárez-Rojas, José Manuel Rodríguez-Pérez, José Manuel Fragoso, Carlos Posadas-Romero

**Affiliations:** 1 Departments of Molecular Biology, Instituto Nacional de Cardiología Ignacio Chávez, Mexico City, Mexico; 2 Cardiovascular Genomics Laboratory, Instituto Nacional de Medicina Genómica (INMEGEN), Mexico City, Mexico; 3 Department of Endocrinology, Instituto Nacional de Cardiología Ignacio Chávez, Mexico City, Mexico; 4 Laboratory of Genomic Medicine, Unit of Research, Hospital Juárez de Mexico, Mexico City, Mexico; Children’s National Medical Center, Washington, UNITED STATES

## Abstract

**Aim:**

The role of interleukin 17A (IL-17A) in the inflammatory process has caused interest in the potential significance of IL-17A as a biomarker for coronary artery disease (CAD). The aim of the present study was to evaluate the role of *IL-17A* gene polymorphisms as susceptibility markers for CAD in the Mexican population.

**Methods:**

Four *IL-17A* gene polymorphisms (rs8193036, rs3819024, rs2275913 and rs8193037) were genotyped by 5’ exonuclease TaqMan assays in a group of 900 patients with premature CAD and 667 healthy controls (with negative calcium score by computed tomography), seeking associations with CAD and other metabolic and cardiovascular risk factors using logistic regression analyses.

**Results:**

No single *IL-17A* polymorphism was associated with premature CAD, however two haplotypes (*CAGG* and *TAGA*) were significantly associated with increased risk of premature CAD (OR = 1.35, 95% CI: 1.00–1.84, P = 0.018 and OR = 2.09, 95% CI: 1.16–3.76, P = 0.003, respectively). Moreover, rs3819024 was associated with increased levels of visceral abdominal fat (P = 0.002) and rs8193036 was significantly associated with risk of central obesity (P = 0.020), hypertriglyceridemia (P = 0.027), and metabolic syndrome (P = 0.027) in the premature CAD group, under dominant models adjusted by age, gender, BMI, smoking history, alcohol consumption, and treatment.

**Conclusion:**

The results suggest that *IL-17A* haplotypes are involved in the risk of developing premature CAD and some *IL-17A* polymorphisms are associated with cardiovascular risk factors in Mexican individuals with premature CAD.

## Introduction

Coronary artery disease (CAD) is a complex multifactorial and polygenic disorder resulting from an excessive inflammatory response to various forms of injurious stimuli to the arterial wall [1–3]. Inflammation is recognized as a major contributor to atherogenesis through adverse effects on lipoprotein metabolism and arterial wall biology [4]. Interleukin-17A (IL-17A) is the most widely studied member of the IL-17 cytokine family. It is mainly produced by T-helper (Th)-17 lymphocytes, but is also secreted by natural killer T (NKT) cells, γδ T cells (γδ-17), cytotoxic CD8^+^ T cells (Tc17), and neutrophils [5–7]. High circulating IL-17A levels have been observed in patients with chronic inflammatory diseases such as rheumatoid arthritis, multiple sclerosis and inflammatory bowel disease, and IL-17A is considered a proinflammatory cytokine [8–11].

Several lines of evidence both in animal models and in humans have shown that IL-17A is involved in atherosclerosis. IL-17A plays a proatherogenic inflammatory role during atherogenesis by promoting monocyte/macrophage recruitment into the aortic wall in the mouse model [12], while the administration of IL-17A neutralizing antibodies to apolipoprotein E (ApoE^−/−^) knockout mice markedly reduced early atherosclerotic lesion area and vulnerability [13]. Moreover IL-17A can increase plaque vulnerability in human specimens [14]. Human coronary artery infiltrating T-cells produce IL-17A and gamma interferon, acting synergistically to induce proinflammatory responses in vascular smooth vessel cells [15]. Other studies have reported the proatherogenic effect of IL-17A [16,17]. These findings have suggested that IL-17A might play a role in the development of cardiovascular diseases.

The *IL17A* gene is mapped on the chromosome 6 and presents several polymorphisms [18]. Some of these polymorphisms have been associated with risk of developing several disease including cancer, gastro-duodenal disease, chronic periodontal disease, and inflammatory bowel disease [19–22]. To our knowledge there is only one report of an association of *IL-17A* gene variation and coronary artery disease, found in the Han Chinese population [23]. Gene variation association studies may vary among populations due to genetic differences, including differences in allele frequencies and linkage disequilibrium (LD) structures. Therefore, it is important to examine multiple ethnic populations for the identification of ethnicity-specific loci as well as common susceptibility loci. Considering the important role of the IL-17A cytokine in the development of atherosclerosis and the lack of studies of the role of polymorphisms of this gene in coronary artery disease, the aim of the present study was to analyze if *IL-17A* gene polymorphisms are associated with premature coronary artery disease (CAD) in a case-control association study (GEA or Genetics of Atherosclerotic Disease).

## Material and Methods

### Subjects

All participants provided written informed consent. The study complies with the Declaration of Helsinki and was approved by the Ethics Committee of the Instituto Nacional de Cardiología Ignacio Chávez (INCICH). The primary aim of the Genetics of Atherosclerotic Disease (GEA) Study is to investigate genetic factors associated with premature CAD, and other coronary risk factors in the Mexican population. All GEA participants are unrelated and of self-reported Mexican-Mestizo ancestry (three generations). A total of 1853 individuals were included in the study, 900 diagnosed with premature CAD and 953 apparently healthy controls. Premature CAD was defined as history of myocardial infarction, angioplasty, revascularization surgery or coronary stenosis >50% on angiography, diagnosed before age 55 in men and before age 65 in women. Controls were apparently healthy asymptomatic individuals without family history of premature CAD, recruited from blood bank donors and through brochures posted in Social Services centers. Exclusion criteria for controls included congestive heart failure, liver, renal, thyroid or oncological disease. The selection of the patients and controls of the GEA study were described in a previous study [24]. Demographic, clinical, anthropometric, biochemical parameter and cardiovascular risk factors were evaluated in both patients and controls. Anthropometric parameters were measured by trained personnel and included waist circumference and body mass index (BMI) calculated as weight in kilograms divided by height in square meters. Blood pressure was measured three different times by sphygmomannometry and the average of the last two measurements was obtained. Obesity was defined as BMI ≥30 kg/m2. Central obesity, hypoalphalipoproteinemia, hypertriglyceridemia, and metabolic syndrome were defined using Adult Treatment Panel III (ATP-III) criteria 2002 [25]. Hypercholesterolemia was defined as total cholesterol (TC) levels ≥200 mg/dL. Hypertension was defined as systolic blood pressure ≥140 mmHg and/or diastolic blood pressure ≥90 mmHg or the use of oral antihypertensive therapy. Type 2 Diabetes mellitus (T2DM) was diagnosed according to World Health Organization criteria.

### Computed Tomography of the Chest and Abdomen

Computed tomography of the chest and abdomen were performed using a 64-channel multi-detector helical computed tomography system (Somatom Sensation, Siemens) and interpreted by experienced radiologists. Scans were read to assess and quantify the following: 1) Coronary artery calcification (CAC) score using the Agatston method [26]; 2) total abdominal, subcutaneous and visceral adipose tissue areas as described by Kvist et al. [27] in order to calculate visceral to subcutaneous adipose tissue ratio (VAT/SAT); and 3) hepatic to splenic attenuation ratio (LSAR) as described by Longo et al. [28]. The tomography was performed in all the patients and healthy controls. However, 286 of apparently healthy individuals were positive for CAC (CAC score >0) and were considered as individuals with subclinical atherosclerosis (SA). These individuals were excluded from the case-control analysis, and the control group only included individuals with negative CAC (n = 667).

### Genetic analysis

Genomic DNA from whole blood containing EDTA was isolated by standard techniques. The rs8193036, rs3819024, rs2275913 and rs8193037 *IL-17A* single nucleotide polymorphisms (SNPs) were genotyped using 5’ exonuclease TaqMan genotyping assays on an ABI Prism 7900HT Fast Real-Time PCR system, according to manufacturer’s instructions (Applied Biosystems, Foster City, CA, USA).

### Statistical analysis

All calculations were performed using SPSS version 18.0 (SPSS, Chicago, Il) statistical package. Means ± SD and frequencies of baseline characteristics were calculated. Chi-square tests were used to compare frequencies and ANOVA and Students t-test were used to compare means. ANCOVA was used to determine associations between the polymorphisms and metabolic variables, adjusting by age, gender, BMI, smoking history, alcohol consumption, and treatment, as appropriate. Logistic regression analysis was used to test for associations of polymorphisms with premature CAD under different inheritance models. Genotype frequencies did not show deviation from Hardy-Weinberg equilibrium (P > 0.05). Pairwise linkage disequilibrium (LD, D′) estimations between polymorphisms and haplotype reconstruction were performed with Haploview version 4:1 (Broad Institute of Massachusetts Institute of Technology and Harvard University, Cambridge, MA, USA).

### Functional prediction analysis

We predicted the potential effect of the *IL-17A* SNPs using bioinformatics tools, including FastSNP [29], SNP Function Prediction (http://snpinfo.niehs.nih.gov/snpfunc.htm), Human-transcriptome Database for Alternative Splicing (http://www.h-invitational.jp/h-dbas/), Splice Port: An Interactive Splice Site Analysis Tool (http://www.spliceport.cs.umd.edu/SplicingAnalyser2.html), ESE finder (http://rulai.cshl.edu/cgi-bin/tools/ESE3/esefinger.cgi), HSF (http://www.umd.be/HSF), and SNPs3D (http://www.snps3d.org/).

## Results

General characteristics of the population are shown in Tables 1 and 2.

**Table 1:** Demographic characteristic of the population.

		**Premature CAD (n = 900)**			**Control (n = 667)**			P value *
		**P25**	**Median**	**P75**	**P25**	**Median**	**P75**	
Age (years)		49	54	58	47	52	58	0.011
Body Mass Index (Kg/m^2^)		26	28.33	31.3	25.41	27.96	30.98	0.012
Waist circumference (cm)		91.13	97.25	105.38	84.9	93.5	101.1	<0.0001
Total Abdominal Fat (cm^2^)		332.25	422	521.75	332	439	541	0.121
Subcutaneous Abdominal Fat (cm^2^)		185	241.5	310.75	209	281	370	<0.000
Visceral Abdominal Fat (cm^2^)		124.25	168	215.75	101	141	185	<0.0001
Visceral/Subcutaneous adipose tissue ratio		0.5	0.69	0.918	0.346	0.484	0.663	<0.0001
Blood Pressure (mmHg)	Systolic	107.33	116.33	128.25	106.33	116.33	127.33	0.318
	Diastolic	66.33	72	78.92	66.67	72	78.33	0.692
Heart Rate (bpm)		57.42	64.67	72.67	59.33	65.33	71	0.531
Gender *n* (%)	Male	253 (37.9)			744 (82.6)			<0.0001
	Female	414 (62.1)			156 (17.4)			
Obesity *n* (%)	Yes	209 (31.3)			323 (35.8)			0.034
Central obesity *n* (%)	Yes	529 (79.3)			753 (83.6)			0.016
Tobacco smoking *n* (%)	Current	212 (31.7)			579 (64.3)			<0.0001
	Former	151 (22.6)			114 (12.6)			<0.0001
Use of alcohol *n* (%)	Yes	492 (73.7)			500 (55.5)			<0.0001
Hypertension *n* (%)	Yes	195 (29.2)			602 (66.8)			<0.0001
Hypertensive Medication *n* (%)	Yes	68 (10.2)			812 (90.2)			<0.0001

Data are expressed as median and percentiles 25 and 75.

*P values were estimated using Mann-Whitney U-test continuous variables and Chi-square or Fisher test for categorical values.

**Table 2:** Comparison of biochemical parameters in individuals with premature coronary artery disease and controls.

		**Premature CAD (n = 900)**			**Control (n = 667)**			P value *
		**P25**	**Median**	**P75**	**P25**	**Median**	**P75**	
Total cholesterol (mg/dl)		134.13	161.95	195.08	168	190.2	209.4	<0.0001
HDL-C (mg/dl)		33	38.5	45.48	37.3	46.8	56.9	<0.0001
LDL-C (mg/dl)		70	91.7	117.1	95.88	115.27	132.88	<0.0001
Triglycerides (mg/dl)		119.3	165.25	226	106	141.5	195.8	<0.0001
ApoA1 (mg/dl)		101.6	119.4	136.4	112.8	131.3	157.6	<0.0001
ApoB (mg/dl)		63	79	102	71	86	106	<0.0001
Glucose (mg/dl)		86.25	95	118	84	90	97	<0.0001
HOMA-IR		3.08	4.69	7.35	2.65	3.96	5.84	<0.0001
Alanine transaminase (IU/L)		18	25	36	17	23	32	0.002
Aspartate transaminase (IU/L)		22	26	31.75	20	25	30	0.007
Alkaline Phosphatase (IU/L)		63	76	94	68	81	98.01	<0.0001
Gamma-glutamyl transpeptidase (IU/L)		23	33	50	16	24	42	<0.0001
TC > 200 mg/dL *n* (%)	Yes	235 (35.2)			197 (21.8)			<0.0001
Hypo a-lipoproteinemia *n* (%)	Yes	334 (50.1)			503 (55.8)			0.013
Hypertriglyceridemia *n* (%)	Yes	302 (45.3)			527 (58.5)			<0.0001
Statin and/or Fibrate treatment *n* (%)	Yes	26 (3.9)			138 (15.3)			<0.0001
Type 2 Diabetes Mellitus *n* (%)	Yes	68 (10.2)			326 (36.2)			<0.0001
Metabolic Syndrome *n* (%)	Yes	274 (41.1)			407 (45.2)			0.056

Data are expressed as median and percentiles 25 and 75.

*P values were estimated using Mann-Whitney U-test continuous variables and Chi-square or Fisher test for categorical values.

### Association of polymorphisms with premature CAD

The distribution of the studied polymorphisms was similar in premature CAD patients and healthy controls under all inheritance models tested (Table 3), and no associations of single SNPs with CAD were observed.

**Table 3:** Associations of *IL17A* polymorphisms with premature CAD.

Polymorphism	Alleles*	MAF^a^	MAF^a^	Genotypes	Genotypes	P_hwe_	OR (95% CI)
		CAD	Control	Premature CAD	Control	CAD/Control	P_dom_ value
rs8193036	*T/C*	0.21	0.22	546/303/46	409/221/34	0.63/0.57	0.99 (0.77–1.26); 0.91
				0.61/0.34/0.05	0.61/0.33/0.05		
rs3819024	*A/G*	0.17	0.19	615/259/24	434/209/24	0.72/1.00	0.87 (0.68–1.12); 0.29
				0.68/0.28/0.03	0.65/0.31/0.04		
rs2275913	*G/A*	0.16	0.19	616/259/23	439/202/26	0.55/0.62	0.91 (0.70–1.17); 0.44
				0.69/0.29/0.03	0.66/0.30/0.04		
rs8193037	*G/A*	0.08	0.07	763/126/9	578/83/6	0.13/0.17	1.14 (0.81–1.59); 0.46
				0.85/0.14/0.01	0.87/0.12/0.01		

Adjusted for age, gender, BMI, hypertension, diabetes mellitus, smoking history, TC, HDL-C, LDL-C and triglycerides.

^a^: MAF, minor allele frequency.

CAD: Coronary artery disease.

Phwe: p value from Hardy-Weinberg equilibrium tests.

NS: Not significant.

*Underlined letter denotes the minor allele in the control samples.

Only the dominant model is showed.

### Association of the polymorphisms with metabolic cardiovascular risk factors and metabolic parameters

The effect of the polymorphisms on various metabolic parameters and cardiovascular risk factors was analyzed separately in the premature CAD and control (CAC score = 0) groups. No associations were observed in controls; however, associations with some metabolic parameters were observed in the premature CAD group. The rs8193036 polymorphism was associated with increased risk of central obesity (OR = 1.67, 95% CI: 1.14–2.45, P_dom_ = 0.020), metabolic syndrome (OR = 1.38, 95% CI: 1.05–1.81, P_dom_ = 0027) and decreased risk of hypertriglyceridemia (OR = 0.75, 95% CI: 0.56–0.97, P_dom_ = 0.027), all under a dominant model adjusted by age, gender, BMI, smoking history, alcohol consumption, and treatment (Table 4). Moreover, the rs3819024 polymorphism was significantly associated with increased levels of visceral abdominal fat (OR = 1.003, 95% CI: 1.002–1.005, P = 0.002) and the rs8193037 polymorphism was associated with an increased risk of metabolic syndrome (OR = 1.68, 95% CI: 1.16–2.43, P_dom_ = 0.005) under dominant models adjusted by the same variables (data not shown).

**Table 4:** Associations of the rs8193036/*IL17A* variant with metabolic risk factors in premature CAD individuals.

	***TT* Genotype (n = 546)**	***TC* + *CC* Genotypes (n = 349)**	**OR (95% CI)**	**P value**
Obesity (%)	0.349	0.369	NS	-
Central Obesity (%)	0.809	0.876	1.67 (1.14–2.45)	0.020
Hypo a-lipoproteinemia (%)	0.571	0.538	NS	-
Hypercholesterolemia (%)	0.236	0.191	NS	-
Hypertriglyceridemia (%)	0.613	0.541	0.75 (0.56–0.97)	0.027
Metabolic Syndrome (%)	0.421	0.501	1.38 (1.05–1.81)	0.027
Type 2 diabetes Mellitus (%)	0.384	0.352	NS	-

All associations were tested using logistic regression adjusting for age, gender, BMI, smoking, alcohol consumption and treatment.

(n) Represent the number of cases with each trait.

NS: Not significant.

### Haplotype analysis and SNP functional prediction

The *IL-17A* polymorphisms were in high linkage disequilibrium (D’>0.8 and r2>0.9) and 10 different haplotypes were observed. Two of these haplotypes (H3: *CAGG* and H6: *TAGA*) were significantly associated with increased risk of premature CAD (OR = 1.35, 95% CI: 1.00–1.84, P = 0.018 and OR = 2.09, 95% CI: 1.16–3.76, P = 0.003, respectively) (Table 5).

**Table 5:** *IL17A* haplotypes frequencies in premature CAD and healthy controls.

**Haplotype**	**Block**	**Premature CAD**	**Control**	**OR (95% CI)**	**P value**
H1	*T-A-G-G*	0.614	0.624	NS	-
H2	*T-G-A-G*	0.095	0.105	NS	-
H3	*C-A-G-G*	0.103	0.078	1.35 (1.00–1.84)	0.018
H4	*C-A-G-A*	0.046	0.054	NS	-
H5	*C-G-A-G*	0.045	0.052	NS	-
H6	*T-A-G-A*	0.035	0.017	2.09 (1.16–3.76)	0.003
H7	*T-G-G-G*	0.021	0.025	NS	-
H8	*C-A-A-G*	0.017	0.022	NS	-
H9	*T-A-A-G*	0.015	0.011	NS	-
H10	*C-G-G-G*	0.011	0.011	NS	-

CD, coronary artery disease; OR, odds ratio; CI, confidence interval; NS, not significant. The order of the polymorphisms in the haplotypes is according to the positions in the chromosome (rs8193036, rs3819024, rs2275913, rs8193037).

Based on SNP functional prediction software, the rs8193036 polymorphism seems to be functional. The presence of the *C* allele in this polymorphism produces a DNA binding site for GATA-3 transcription factors with possible consequences on IL-17A expression.

## Discussion

IL-17A is a pleiotropic cytokine with effects on multiple cell types to enhance the production of proinflammatory molecules and several lines of evidence have shown it is involved in the pathogenesis of atherosclerosis [12, 15–17, 30]. In spite of this known role of the IL-17A in the development of atherosclerosis, only one study has explored whether *IL-17A* gene variation plays a role in susceptibility to coronary artery disease. Zhang et al. [23] studied 5 *IL-17A* polymorphisms (rs4711998, rs3819024, rs2275913, rs8193037, and rs3819025) in 1031 CAD patients and 935 healthy controls, reporting a significant association of rs8193037 with CAD in the Chinese Han population. In our study, this polymorphism was not associated with premature CAD, but was associated with metabolic syndrome in the premature CAD group. Moreover, rs3819024 was associated with increased levels of visceral abdominal fat and rs8193036 (not studied by Zhang et al.) was associated with risk of central obesity, metabolic syndrome and hypertriglyceridemia in Mexican CAD patients. It is important to note that Zhang et al. (2011) did not examine the association between these polymorphisms and metabolic parameters or other cardiovascular risk factors, and these observations must be confirmed in other studies.

The functional software used here predicted that rs8193036 as a functional polymorphism. The presence of the rs8193036 *C* allele produces a DNA binding site for the transcription factors GATA-3 with possible consequences increasing IL-17A expression and its pro-inflammatory effects. The increased inflammation could affect various metabolic parameters and cardiovascular risk factors, explaining the associations observed in the premature CAD group. Although the rs8193036 functional prediction is in agreement with our observations, the results must be interpreted with caution as we did not perform expression analysis and have no evidence that the IL-17A expression is in fact different in premature CAD patients with the risk allele.

In our study, the *IL-17A* polymorphisms were in strong linkage disequilibrium, and two of the haplotypes (H3: *CAGG* and H6: *TAGA*) were associated with premature CAD. To our knowledge, there are no previous studies analyzing associations of these haplotypes with CAD or any other disease.

Study limitations need to be addressed. In this study, only four *IL-17A* polymorphisms were analyzed. Since this is the first study reporting associations of *IL-17A* polymorphisms with metabolic parameters and cardiovascular risk factors, replication in other groups of patients is necessary to confirm our results. The predicted functional consequences of the rs8193036 polymorphism using informatics tools need to be corroborated by experimental evidence. A positive point of our work is that the control group only included individuals without subclinical atherosclerosis (individuals without coronary artery calcification).

## Conclusions

In summary, our study demonstrates the association of some *IL-17A* polymorphism with metabolic parameters and cardiovascular risk factor in a group of Mexican CAD patients. The four *IL-17A* polymorphisms were in high linkage disequilibrium and two haplotypes were significantly associated with premature CAD risk. According to the informatics software, the rs8193036 polymorphism found to be associated with central obesity, metabolic syndrome and hypertriglyceridemia is predicted to have a functional effect. Because the Mexican population has a distinct genetic background [31–34], the *IL-17A* polymorphism associations observed here should be explored in other populations.
